# Disaster experience and resident risk preference: Evidence from China household finance survey

**DOI:** 10.1371/journal.pone.0295146

**Published:** 2023-11-30

**Authors:** Linan Guo, Weiming He, Jiaxue Wang

**Affiliations:** Institute of Disaster Prevention, Sanhe, China; National Technical University of Athens: Ethniko Metsobio Polytechneio, GREECE

## Abstract

China is one of the countries hardest hit by disasters. Disaster shocks not only cause a large number of casualties and property damage but also have an impact on the risk preference of those who experience it. Current research has not reached a consensus conclusion on the impact of risk preferences. This paper empirically analyzes the effects of natural and man-made disasters on residents’ risk preference based on the data of the China Household Financial Survey (CHFS) in 2019. The results indicate that: (1) Both natural and man-made disasters can significantly lead to an increase in the risk aversion of residents, and man-made disasters have a greater impact. (2) Education background plays a negative moderating role in the impact of man-made disasters on residents’ risk preference. (3) Natural disaster experiences have a greater impact on the risk preference of rural residents, while man-made disaster experiences have a greater impact on the risk preference of urban residents. Natural disaster experiences make rural residents more risk-averse, while man-made disaster experiences make urban residents more risk-averse. The results provide new evidence and perspective on the negative impact of disaster shocks on the social life of residents.

## 1 Introduction

Disasters are usually divided into two categories according to their causes: natural disasters and man-made disasters. Both of them have brought a large number of casualties and property losses around the world. Furthermore, disaster experiences may also affect an individual’s perspective, mood, and risk preference [1, 2].

Risk preference is defined as the psychological attitude of decision-makers toward risks. It is important for saving, consumption, investment, and other behaviors. People’s risk preference may change under the influence of many factors, such as income [3, 4], age [5], educational background [6], and so on, and similarly, disaster shocks can have certain effects on individuals preference [7]. Currently, there is still a debate about the direction and extent of the impact of disasters on the risk preference of the population.

As to natural disasters, some studies suggest that more residents tend to be risk-averse after experiencing the impact of natural disasters [8, 9], even without significant personal losses [10]. Zhang et al. [11], based on the urban household survey data of the National Bureau of Statistics of China, found that the earthquake impact significantly made urban households more risk-averse. The closer the families were to the epicenter, the more the probability and expenditure of buying lottery tickets after the earthquake decreased. And other studies have reached different conclusions. Page et al. [12] studied changes in individuals’ attitudes towards risk after a disaster in the context of the 2011 Australian floods and found that individuals who suffered losses were willing to accept higher risks after the disaster to obtain higher rewards to compensate for the economic losses caused by the floods. Eckel et al. [13] studied the risk preference of evacuees during Hurricane Katrina and also reached consistent conclusions. Furthermore, Callen [14] found no changes in the risk preference of Sri Lankan workers after the 2004 Asian tsunami. Bchir and Willinger [15] found that low-income individuals exposed to areas of mudslide risk were more likely to take risks through a risk preference study of individuals experiencing mudslide risk and that the risk preference of high-income individuals was not significantly affected. Hanaoka et al. [16] took the 2011 earthquake in Japan as the cut-off point to study whether human risk preference changed before and after the earthquake. The results showed that men were more likely to seek risks after the earthquake, and the effect lasted longer. In addition, climate change and wildfire disasters caused by hot weather will also have an impact on the lives of residents and stakeholders [17, 18], which will prompt action by stakeholders accordingly [19]. Hand [20] tested public agency wildfire managers in the context of wildfires and reached the same conclusion.

As to man-made disasters, it refers to disasters caused by human factors, such as wars, terrorist violence, car accidents, shipwrecks, nuclear accidents, and so on. [21]. There is relatively little research on the impact of man-made disasters on risk preference. By analyzing investor attitudes before and after the 2008 financial crisis, Guiso et al. [22] found that the risk-averse behavior of investors increased even though some investors did not suffer any losses during the crisis. By examining the risk attitudes of residents in Ukraine and Germany during the Great Recession, Dohmen et al. [23] found an increase in risk aversion and a significant decrease in willingness to take risks in both countries during this period. Some studies have also found that individuals who experienced violent events (including terrorist attacks, civil wars, and so on.) are more risk-averse [24–26], while Voors et al. [7] studied individual risk preference after the civil war in Burundi and came to the opposite conclusion that individuals who experienced violent events individuals are more risk-seeking.

After reviewing the above literature, it is found that scholars mainly focus on natural disasters in terms of the impact of exogenous shocks on risk preference, and almost no comparative studies have been conducted on the impact of natural and man-made disasters on residents’ risk preference under a unified framework. Meanwhile, in addition to single disasters, overall annual disasters can also cause serious losses, so does it also have an impact on residents’ risk preference? In addition, it remains to be explored whether there are differences in the direction, degree, and path of impact of the two types of disasters on residents’ risk preference.So we analyze the effects of natural and man-made disasters on residents’ risk preference based on the data from the 2019 China Household Financial Survey (CHFS) questionnaire, reveal the differences in the impacts of the two types of disasters on residents and the reasons for them, and further discuss the main channels through which disaster experiences affect residents’ risk preference by grouping residents. We expand the research on the impacts of disaster experiences on risk preferences in the existing literature by analyzing natural and man-made disasters in the same framework. With the frequency of disasters, understanding and grasping the impacts and mechanisms of the negative shocks of different disasters on residents’ risk preference is crucial for effective economic recovery after disasters.

The remainder of this paper is organized as follows. In the next section, we formulate the research hypotheses. Section 3 follows with the research design. The empirical analysis and robustness tests are then performed to identify the disaster shocks that affect residents’ risk preference. Section 5 shows the extension and further discussion of the model. Section 6 contains the conclusions and recommendations.

## 2 Research hypothesis

Risk perception is a concept used to describe people’s attitudes and intuitive judgments about risk [27]. Generally, individuals make subjective risk judgments based on experiences gained from intuitive judgments and subjective feelings and combine them with environmental stimuli as a basis for behavioral decisions [28]. Disaster shocks can cause casualties and property damage, which will bring indelible psychological shadows to those who experience them. Any negative emotions generated by those who experience them will significantly affect their level of risk perception and change their risk preference [1]. According to the conclusions of the literature, the impact of natural disasters on residents’ risk preference is not completely consistent. However, based on the domestic research results, we find that earthquake disasters make residents more risk-averse [11]. The impact of “major accidents” can significantly reduce individuals’ risk-seeking [29]. Since the impact of man-made disasters is similar to that of natural disasters, we believe that both natural disasters and man-made disasters bring negative effects, and the risk perception of those who experience them may be changed and their risk aversion will be more intense. Accordingly, we propose the following hypothesis:

**Hypothesis 1: Both natural and man-made disaster experiences make residents more risk-averse.**

Due to the different occurrence characteristics and impact degrees of natural disasters and man-made disasters, the risk perception of residents may be different, which in turn has different effects on risk preference. Firstly, residents will get help from the government and the community after natural disasters, which will increase their trust in the outside world [10] and increase their confidence in the government to some extent, while relief after a man-made disaster event is often perceived as disguised risk compensation [30, 31], which may lead to higher levels of expected risk and distrust of the outside world among residents.

Secondly, from the perspective of post-disaster psychology, when natural disasters break out, residents’ behaviors will evolve into group behaviors based on common disaster impact and consistent post-disaster psychology, and the influence degree of their risk preference may be weakened. As for man-made disasters, most of them are individual disasters with strong destructiveness but a narrow scope of influence. This is different from group disasters represented by natural disasters. Individual disasters may be more risk-averse due to the “psychological gap” after the disaster. In summary, we proposes that:

**Hypothesis 2: The impact of natural disasters on residents’ risk preference is weaker than that of man-made disasters.**

## 3 Research design and sampling

### 3.1 Data source

The data is the micro household data in the China Household Financial Survey database (Hereinafter referred to as CHFS.), collected in 2019 by the Southwestern University of Finance and Economics in China. The CHFS conducts a random national sample every two years, covering the entire country. To reduce the interference with the results, the samples are processed as follows: (1) Eliminate samples with missing key indicators; (2) Eliminate the samples that cannot directly judge the risk preference of residents; (3) Considering the characteristics of venture capital participation groups and referring to the way Liu and Tian [32] handle the data, the samples of respondents under 18 years old and over 80 years old are eliminated, and finally 26,475 observed values are obtained, distributed in 29 provinces (municipalities directly under the Central Government).

### 3.2 Variable settings

We set residents’ risk preferences as the dependent variable. In previous literature, the measurement of risk preference mainly adopts residents’ subjective answers to a question that can reflect residents’ risk preference and is easy to answer, such as investment choice preference. Similarly, in the CHFS survey, one of the questions the respondents were asked is “If you have a sum of money for investment, which investment project would you most like to choose?”. 5 options can be chosen, which are A: high risk, high return project, B: Slightly high risk, slightly high return project, C: average risk, average return project, D: slightly lower risk, slightly lower return project, and E: not willing to take any risk. Referring to the index design method in Xu et al. [29], subjective responses to this question are treated as risk preference indicators in this paper, and the risk preference values are set 4, 3, 2, 1, and 0 corresponding to each option, “4” represent the highest risk seeking and “0” represent the highest risk aversion. So the risk preference is an ordered limited discrete variable.

The main independent variables are natural and man-made disaster experiences. We used a question from the CHFS questionnaire to describe natural and man-made disaster experiences. It’s a multiple-choice question. The question is: “Since 2014, has there been at least one event in your household that had a significant impact on you?” There are three options available for answering, among which, A: My family has experienced some natural disasters (e.g., earthquakes, floods, tsunamis, etc.), B: My family has experienced some man-made disasters (e.g., fires, car accidents, etc.), and C: None. To discriminate the effects of natural and man-made disasters. Control variables generally contain individual basic characteristics and household factors. Based on previous studies, we use six indicators to represent the basic characteristics of individuals: age, gender, educational background, marital status, degree of economic attention, and happiness. Considering that residents’ age may have a non-linear influence on their behavior, the squared term variable of age is introduced with reference to the study of Yu et al. [33]. Four family indicators are considered, which are household registration type, whether or not purchased commercial insurance, the logarithm of total annual household income, and the logarithm of total annual household consumption. The specific variable settings are shown in Table 1.

**Table 1:** Variable definition and statistics.

	Sign	Variable definitions and descriptions
Dependent variable	Risk_preference	Not willing to take any risk = 0 Slightly lower risk, slightly lower return project = 1 Average risk, average return project = 2 Slightly high risk, slightly high return project = 3 High risk, high return project = 4
Main independent variable	Natural_disaster	Not experienced natural disasters = 0 Experienced natural disasters = 1
	Man_Made_disaster	Not experienced man-made disasters = 0 Experienced man-made disasters = 1
Control variable	Gender	Female = 0 Male = 1
	Age	Age of respondents
	Age_sq	Squared term of age (Age^2^/100)
	Education	Uneducated = 0 Primary school = 1 Junior high school = 2 Senior high school = 3 Technical secondary school/vocational school = 4 Junior college/higher vocational school = 5 Bachelor degree = 6 Master degree = 7 Doctoral degree = 8
	Marriage	Unmarried = 0 Married = 1
	Attention	Never pay attention to economics and finance = 0 Seldom pay attention to economics and finance = 1 Sometimes pay attention to economics and finance = 2 Often pay attention to economics and finance = 3 Always pay attention to economics and finance = 4
	Happiness	Very unhappy = 0, Unhappy = 1 Average = 2, Happy = 3, Very happy = 4
	Area	Rural (Household registered in rural areas) = 0 Town (Household registration in town areas) = 1
	Insurance	Didn’t buy any commercial insurance = 0, Have bought commercial insurance = 1
	Lninc_total	Logarithmic value of respondents’ total annual household income
	Lncon_total	Logarithmic value of total annual consumption of respondents’ households

### 3.3 Descriptive statistics

With reference to the study of Ntanos [34], we made a descriptive statistical analysis of the data, and the results are shown in Table 2. It can be seen that in terms of disaster experience, the vast majority of residents have had no experience of disasters, with only 4% having experienced natural disasters and 1.7% having experienced man-made disasters. In terms of risk preference, the mean subjective risk preference is 0.698, which indicates that most residents are risk averse. In terms of demographic characteristics, the average age of residents is older, about 53 years old. The mean value of education is 2.557, indicating that most residents are at junior high and high school levels. Most residents are married, accounting for 83.8%. The mean value of residents’ economic attention degree is 0.869, indicating that most residents pay little attention to economic and financial information. The mean value of residents’ happiness is 2.848, which means that residents are generally happy with their lives. At the household level, there are more urban residents, accounting for 67.5%. Residents’ insurance awareness is insufficient, and only 11.8% of residents have bought commercial insurance. Total income and total consumption are within a reasonable range overall.

**Table 2:** Descriptive statistics.

Variables	Obs	Mean	Std. Dev.	Min	Max
Risk preference	26475	0.698	1.038	0	4
Natural disaster	26475	0.04	0.197	0	1
Man_made_disaster	26475	0.017	0.128	0	1
Gender	26475	0.522	0.5	0	1
Age	26475	53.406	14.038	18	80
Age sq	26475	30.492	14.513	3.24	64
Education	26475	2.557	1.705	0	8
Marriage	26475	0.838	0.369	0	1
Attention	26475	0.869	1.022	0	4
Happiness	26475	2.848	0.851	0	4
Rural	26475	0.675	0.468	0	1
Insurance	26475	0.118	0.323	0	1
Lninc total	26475	10.726	1.402	0.049	16.311
Lncon total	26475	10.93	0.876	7.712	14.795

### 3.4 Research methodology

Residents’ subjective risk preference is taken as the dependent variable, and residents’ experiences of natural and man-made disasters are taken as the core independent variables. The following model is established to identify the parameters to be estimated:

$${Risk\_preference}_{i}*=\beta_{0}+\beta_{1}\times {Natural\_disaster}_{i}+\beta_{2}\times {Man\_Made\_disaster}_{i}+X_{i}\theta +\mu_{i}$$ (1)

$$Where\;{Risk\_preference}_{i}=\left\{ \begin{array}{l} 0,\;{Risk\_preference}_{i}*\leq r_{0}\\ 1,\;r_{0}<{Risk\_preference}_{i}*\leq r_{1}\\ 2,\;r_{1}<{Risk\_preference}_{i}*\leq r_{2}\\ 3,\;r_{2}<{Risk\_preference}_{i}*\leq r_{3}\\ 4,\;{Risk\_preference}_{i}*>r_{3} \end{array}\right.$$ (2)

Among them, Risk_preference_i_ * is the latent variable of residents’ subjective risk preference. *r*_*0*_*、r*_*1*_*、r*_*2*_*、r*_*3*_*、r*_*4*_ are the intercepts that satisfy *r*_*0*_<*r*_*1*_<*r*_*2*_<*r*_*3*_<*r*_*4*_. Natural_disaster_i_ is the residents’ experience of natural disasters, and Man_Made_disaster_i_ is the residents’ experience of man-made disasters. *X*_*i*_ is the control variable matrix including other important factors, such as residents’ individual and household characteristics. *μ*_*i*_ is the random error term. Due to the ordered characteristics of subjective risk preference, this paper uses the ordered response model to estimate the parameters in Eq (1).

## 4 Empirical results and analysis

### 4.1 Impact of disaster experiences on residents’ risk preference

Models (1)—(2) of Table 3 report the impact of natural and man-made disaster shocks on residents’ risk preference. On this basis, the marginal effects of residents’ natural and man-made disaster experiences on their risk preferences were calculated. Considering the large span of dependent variables (0~4), the marginal effect results based on the ordered Logit model are complicated. In order to facilitate the comparison of the impact degree of natural disasters and man-made disasters on residents’ risk preference, the study of Li and Feng [35] is referred to. In order to determine the marginal effect under different risk preference, three variables of the highest, middle, and lowest risk preference are selected. Specifically, marginal effects corresponding to Risk_preference = 0, 2, and 4 are analyzed. The results reported are models (3)—(5). The results show that: after controlling for individual characteristics and household factors, the regression coefficients of both natural disaster experience and man-made disaster experience are significantly negative, implying that both types of disaster experience make residents more risk averse, so Hypothesis 1 is proved. The finding is also mutually confirmed with the research of Cameron and Shah [8], Chantarat et al. [9], and Wang and Young [24].

**Table 3:** Effects of natural and man-made disasters on risk preference.

Variable	(1) Ordered Logit	(2) Ordered Logit	(3) Risk_preference = 0	(4) Risk_preference = 2	(5) Risk_preference = 4
Natural_disaster	-0.215***	-0.138*	0.025*	-0.012*	-0.004*
	(0.070)	(0.072)	(0.013)	(0.006)	(0.002)
Man-Made_disaster	-0.298***	-0.288***	0.053***	-0.025***	-0.007***
	(0.104)	(0.105)	(0.019)	(0.009)	(0.003)
Gender	0.236***	0.289******	-0.053***	0.025***	0.007***
	(0.026)	(0.027)	(0.005)	(0.002)	(0.001)
Age	-0.056***	-0.057***	0.010***	0.010***	-0.001***
	(0.006)	(0.006)	(0.001)	(0.001)	(0.000)
Age_sq	0.011*	0.015**	-0.003**	-0.003**	0.000**
	(0.006)	(0.006)	(0.001)	(0.001)	(0.000)
Education	0.189***	0.121***	-0.022***	0.010***	0.003***
	(0.009)	(0.009)	(0.002)	(0.001)	(0.000)
Marriage	-0.044	-0.165	0.030***	-0.014***	-0.004***
	(0.038)	(0.039)	(0.007)	(0.003)	(0.001)
Attention	0.521*	0.494***	-0.091***	0.043***	0.013***
	(0.014)	(0.014)	(0.002)	(0.001)	(0.001)
Happiness	-0.057***	-0.054**	0.010***	-0.005***	-0.001***
	(0.016)	(0.016)	(0.003)	(0.001)	(0.000)
Area		0.116***	-0.021***	0.010***	0.003***
		(0.034)	(0.006)	(0.003)	(0.001)
Insurance		0.233	-0.043***	0.020***	0.006***
		(0.036)	(0.007)	(0.003)	(0.001)
Lninc_total		0.050***	-0.009***	0.004***	0.001***
		(0.012)	(0.002)	(0.001)	(0.000)
Lncon_total		0.230***	-0.042***	0.006***	0.006***
		(0.020)	(0.004)	(0.001)	(0.001)
N	26475	26475	26475	26475	26475
R^2^	0.1187	0.1243	0.124	0.124	0.124

Note: Values in parentheses are robust standard errors of regression coefficient estimates

*, **, and *** indicate significance at the 10%, 5%, and 1% levels, respectively, as follows.

Among the effects of control variables, the coefficient of gender is significantly positive, which indicates that men are more risk-seeking than women, which is consistent with the research conclusion of Thomas Dohmen et al. [23]. The coefficient of age is significantly negative in both models, while the coefficient of age squared is significantly positive, that is, age has a significant positive U-shaped effect on risk preference, which means that the willingness of risk-seeking of residents shows a trend of gradual decrease with the increase of age, and this trend gradually disappears with the increase of age. This may be because middle-aged people have lower risk tolerance. After all, they have to support the elderly and children. All other control variables have a significant effect on residents’ subjective risk preference, except for marital status and the purchase of commercial insurance.

Taking into account the marginal effects and assuming all covariates at their respective means, exposure to natural disaster shocks results in a 0.4% reduction in the probability of residents exhibiting a high-risk preference, a 1.2% reduction in the probability of a medium-risk preference, and a concurrent 2.5% increase in the probability of a low-risk preference, as compared to individuals not exposed to such shocks. In contrast, relative to residents not subject to man-made disaster shocks, exposure to these shocks corresponds to a 0.7% decrease in the probability of possessing a high-risk preference, a 2.5% decrease in the probability of a medium-risk preference, and, inversely, a 5.3% increase in the likelihood of a low-risk preference. It can be concluded that the impact of man-made disaster experiences on residents’ risk preference is higher than that of natural disasters. In general, the reported results support hypotheses 1 and 2 presented in this paper.

### 4.2 Robustness tests

In this research, robustness tests of the reported results are done in two ways. The first approach is to change the regression method. To verify that the conclusions of this research are not affected by the regression methods, the Logit model is replaced with OLS and Probit models. The regression results are indicated in Table 4 of Model (1) and Model (2). From the results, it can be seen that both types of disaster experiences have a significant negative effect on risk-seeking willingness, which is consistent with the findings of the baseline regression. The second approach is to use binary risk preference to replace the dependent variables. In Model (1) and Model (2) of Table 3, ordered risk preference is used as the subjective risk preference indicator measure for residents. On this basis, binary risk preference is used as the indicator measure of subjective risk preference, that is, the selection of “projects with slightly high risk and slightly high return” and “projects with high risk and high return” are defined as a high-risk preference and denoted as 1. The other three types of choices are defined as a low-risk preference, denoted as 0. The regression results are shown in Table 4 of the model (3). The results show that both natural disaster experiences and man-made disaster experiences significantly reduce residents’ risk-seeking willingness, and the effects of other control variables on risk preference are also generally consistent with the results reported in Table 4.

**Table 4:** Robustness test results.

Variable	(1) OLS	(2) Probit	(3) Logit (binary)
Natural_disaster	-0.120***	-0.131***	-1.999***
	(0.024)	(0.040)	(0.360)
Man-Made_disaster	-0.150***	-0.195***	-1.683***
	(0.034)	(0.060)	(0.442)
Gender	0.141***	0.177***	0.620***
	(0.012)	(0.016)	(0.057)
Age	-0.048***	-0.034***	-0.036***
	(0.003)	(0.004)	(0.013)
Age_sq	0.029***	0.010***	0.003*
	(0.003)	(0.004)	(0.014)
Education	0.049***	0.060***	0.074***
	(0.005)	(0.005)	(0.019)
Marriage	-0.099***	-0.099***	-0.249***
	(0.017)	(0.023)	(0.078)
Attention	0.219***	0.277***	0.473***
	(0.007)	(0.008)	(0.026)
Happiness	-0.019***	-0.028**	-0.078**
	(0.007)	(0.010)	(0.034)
Area	0.022	0.046**	0.062
	(0.014)	(0.020)	(0.073)
Insurance	0.145	0.136	0.251
	(0.020)	(0.021)	(0.068)
Lninc_total	0.011**	0.022***	-0.004
	(0.005)	(0.007)	(0.024)
Lncon_total	0.091***	0.134***	0.281***
	(0.008)	(0.011)	(0.039)
_cons	0.993***		
	(0.117)		
N	26475	26475	26475
R^2^	0.2248	0.1146	0.1373

## 5 Extension of the model and further discussion

To further explore the differences in residents’ subjective risk preference caused by disasters, we selected gender and educational background from the basic characteristics of residents and household registration type from the household factors and grouped the samples in separate regressions to test the effects of natural and man-made disaster experiences on subjective risk preference. Therefore, the variable Settings and regression methods of all models in this part are the same as those of model (2) in Table 3. The only difference is the sample size.

### 5.1 Sub-samples on gender

Two sub-samples are divided according to the gender of the residents. The regression results are shown in Table 5. The regression coefficients of Natural_disaster in the model (1) and model (2) are both significantly negative, indicating that both men’s and women’s experience of natural disasters has a significant negative impact on their risk-seeking willingness, and that men’s performance is more pronounced, and this result is in line with the conclusions of Zhang et al. [11] in his study of the impact of earthquake shocks on risk preferences. The coefficient of Man_Made_disaster is negative in both models, but only the coefficient in the model (1) is more significant, indicating that experience of man-made disasters has a significant negative effect on women’s risk-seeking willingness, while men’s is not significant. The main reason considered is that women are more emotional than men, and both natural and man-made environmental stimuli may have a more pronounced effect on their risk preference.

**Table 5:** Sub-sample estimates for gender.

Variable	Female (1)	Male (2)
Natural_disaster	-0.228*	-0.201**
	(0.120)	(0.086)
Man_Made_disaster	-0.415**	-0.212
	(0.167)	(0.134)
Control	Yes	Yes
N	12666	13809
R^2^	0.1209	0.1274

### 5.2 Sub-samples on education background

In general, the higher the level of education is, the higher the disaster cognition is, the stronger the ability to discriminate disaster risks is, and the risk preference may also change accordingly. The regression results are shown in Table 6. It can be seen that residents with no educational experience show insignificant risk preference for both types of disaster shocks. Disaster experience has a significant negative effect on the risk preference of residents with primary education, and the significance of the Natural_disaster coefficient is higher than Man_Made_disaster. For the residents with higher education, the experience of natural disasters did not significantly affect their risk preference, but the experience of man-made disasters significantly make them more risk averse. Thus, increased levels of education can alter the extent to which disaster experience affects risk preference. This is consistent with the findings of Joshi et al. [36].

**Table 6:** Sub-sample estimates of educational background.

Variable	Uneducated (1)	Primary Education (2)	Higher Education (3)
Natural_disaster	0.160	-0.177**	-0.196
	(0.215)	(0.076)	(0.295)
Man_Made_disaster	-0.603	-0.217*	-0.772***
	(0.580)	(0.113)	(0.292)
Control	Yes	Yes	Yes
N	1679	19970	4826
R^2^	0.0558	0.0842	0.0970

### 5.3 Sub-samples on household registration type

In terms of natural disasters, compared with urban areas, the terrain of rural areas is more complex, the ecological environment is more fragile, and the ability to resist natural disasters is relatively weak. As for man-made disasters, urban areas with complex building structures and high population density have a higher probability of man-made disasters and cause more serious losses. Thus, the two types of disasters may have different intensities of impacts on rural and urban residents, and their risk preference may be different to some extent. For this reason, two sub-samples of rural and urban residents were divided and regression tests were conducted separately. The results are shown in Table 7. It can be seen that natural disasters make rural residents more inclined to avoid risks, and the impact on urban residents is not obvious. However, the experience of man-made disasters makes urban residents more risk-averse and has no obvious impact on rural residents.

**Table 7:** Sub-sample estimates of household registration types.

Variable	Rural (1)	Town (2)
Natural_disaster	-0.161*	-0.064
	(0.085)	(0.119)
Man_Made_disaster	-0.184	-0.326**
	(0.156)	(0.139)
Control	Yes	Yes
N	8606	17869
R^2^	0.0669	0.1368

## 6 Conclusion and recommendations

This paper empirically examines the effects of two types of disaster shocks on residents’ subjective risk preference, using natural disaster experiences and man-made disaster experiences as exogenous shocks in 2019 in China. Overall, both natural disaster shocks and man-made disaster shocks significantly increased residents’ risk aversion, and the effect of man-made disaster shocks is more significant. The sub-sample analysis shows that: (1)Women’s experience of disasters affects their subjective risk preferences to a greater extent than men’s; (2) Education background plays a negative moderating role in the impact of man-made disasters on residents’ risk preference; The impact of the two types of disasters experiences on residents’ risk preference in rural and urban areas differs. Natural disaster experience has a greater impact on the risk preference of rural residents, while man-made disaster experience has a greater impact on the risk preference of urban residents.

In order to reduce the impact of disasters on residents, the government should, on the one hand, strengthen the monitoring and early warning ability of natural disaster risks, timely release disaster information, and reduce the disaster losses of residents. On the other hand, they should actively carry out pilot projects of natural disaster insurance in rural areas, strengthen the coverage of man-made disaster insurance in urban commercial areas, such as traffic hazard insurance and fire insurance, and encourage residents to buy catastrophe insurance to provide more comprehensive and effective risk protection for urban and rural residents. In addition, financial institutions should also adjust the types and pricing strategies of their products and services promptly according to the changes in residents’ risk preference to meet the post-disaster needs of different residents.

## 7 Limitations and future research

In this paper, only one single year of CHFS survey data is used to start the analysis of residents’ risk preference, and the existing results can reflect the performance at that time to a certain extent, but with the passage of time, the residents’ risk preference may also be affected by the continuous development of the society. If it can be analyzed by combining the surveys of previous years to form the panel data, the results may be more representative.

Future research could also consider further refining the disaster experience, from earthquakes, floods, droughts, traffic accidents, fires and other disasters separately, which would be more interesting. Also, considering the disaster effects on stakeholders will be an interesting research too.
